# Screening for Optimal Liposome Preparation Conditions by Using Dual Centrifugation and Time-Resolved Fluorescence Measurements

**DOI:** 10.3390/pharmaceutics13122046

**Published:** 2021-11-30

**Authors:** Jonas K. Koehler, Johannes Schnur, Heiko Heerklotz, Ulrich Massing

**Affiliations:** 1 Institute of Pharmaceutical Sciences, University of Freiburg, 79104 Freiburg im Breisgau, Germany; johannes.schnur@posteo.de (J.S.); heiko.heerklotz@pharmazie.uni-freiburg.de (H.H.); 2 Signaling Research Centers BIOSS and CIBSS, University of Freiburg, 79085 Freiburg im Breisgau, Germany; 3 Leslie Dan Faculty of Pharmacy, University of Toronto, Toronto, ON M5S 3M2, Canada; 4 Andreas Hettich GmbH & Co. KG, 78523 Tuttlingen, Germany

**Keywords:** dual centrifugation, liposomes, vesicular phospholipid gel, encapsulation efficiency, time-resolved fluorescence, differential scanning calorimetry

## Abstract

Dual centrifugation (DC) is a novel in-vial homogenization technique for the preparation of liposomes in small batch sizes under gentle and sterile conditions which allows encapsulation efficiencies (*EE*) for water soluble compounds of >50%. Since liposome size, size distribution (PDI), and *EE* depend on the lipid concentration used in the DC process, a screening method to find optimal lipid concentrations for a defined lipid composition was developed. Four lipid mixtures consisting of cholesterol, hydrogenated or non-hydrogenated egg PC, and/or PEG-DSPE were screened and suitable concentration ranges could be identified for optimal DC homogenization. In addition to the very fast and parallel liposome preparation of up to 40 samples, the screening process was further accelerated by the finding that DC generates homogeneously mixed liposomes from a macroscopic lipid mixture without the need to initially prepare a molecularly mixed lipid film from an organic solution of all components. This much simpler procedure even works for cholesterol containing lipid blends, which could be explained by a nano-milling of the cholesterol crystals during DC homogenization. Furthermore, *EE* determination was performed by time-resolved fluorescence measurements of calcein-loaded liposomes without removing the non-entrapped calcein. The new strategy allows the rapid characterization of a certain lipid composition for the preparation of liposomes within a working day.

## 1. Introduction

Dual centrifugation (DC) was first described by Massing et al. in 2007 [1] as a powerful method for the preparation of vesicular phospholipid gels (VPGs) and, after simple dilution of a VPG, of a liposomal dispersion. DC homogenization of viscous lipid/buffer mixtures along with ceramic beads in small disposable vials is achieved by the additional, fast turning of the sample vial around its own axis (axis: 90° with respect to the longitudinal direction of the vial) during normal centrifugation. This results in a frequent and very powerful movement of the viscous sample material from top to bottom of the vial and vice versa. Strong bead–bead interactions take place and the sample material in between the beads becomes homogenized, particularly when the lipid mixture and the cloud of beads reach the distal regions of the vials (cloud homogenization). This very powerful movement of the potentially highly viscous samples is based on the high centrifugal acceleration of up to 1000× *g* caused by the rotation around the primary axis, and is more than one magnitude higher than the sample acceleration that can be reached by the most powerful lab shakers [2].

A more traditional method to prepare VPGs has been high-pressure homogenization (HPH) [3]. DC produces VPGs and liposomes that are very similar to those made by high-pressure homogenization but does so in a gentler way. This results from the quasi-continuous action of comparatively moderate shear forces in the DC compared to a limited number of extreme-shear cycles in HPH. This way, DC reduces the risk of lipid breakdown products such as lysophosphatidylcholine and allows the entrapment of sensitive drugs. Additionally, the rather long duration of the DC process (typically 15–30 min for liposomes) makes it possible to efficiently cool the samples during DC [2], which allows the entrapment of sensitive proteins [4,5].

DC allows the simultaneous preparation of up to 40 samples in one run. The liposomes can be prepared sterile and in very small batch sizes in closed vials [1], which is especially advantageous when small batches of liposomes are needed (e.g., for biological tests) or when the lipids or the drug compound are rare and/or expensive. Preparations of extremely small amounts of sterile siRNA-containing liposomes by DC (1 mg) with high entrapping efficiency were reported [6,7]. While both DC and HPH yield equal VPGs and liposomes, small-scale development favors the smaller individual batch sizes possible with DC whereas HPH may facilitate the scale-up to big batches for production.

Shelf life is a major issue in the development of liposomal formulations. DC provides two possible solutions to this problem. First, VPGs prepared by DC can be used as a more stable storage form and can be diluted to form liposomes right before administration [8,9]. When VPGs are stored, there is no loss of encapsulation because there is no concentration gradient between the internal and external aqueous phase of the vesicles [10]. Second, liposomes can be produced by DC in the hospital pharmacy immediately before the administration. To this end, the aqueous medium can be added to sterile containers, which can be stored including the active pharmaceutical ingredient (API), ceramic beads, and the lipid mixtures to be subjected to DC [1,2]. These special advantages of liposome preparation by DC also had direct impact on the design of our study. Whereas the ability to retain their cargo over significant storage times is a key parameter for most liposomal formulations, it is not of primary importance for DC liposomes and has not been addressed here.

Determination of *EE* after liposome production using a fluorescence dye like calcein typically requires the removal of the non-trapped fluorescence dye, e.g., by centrifugation, dialysis, or column separation [11]. Due to the mechanical stress caused by the separation, liposome sizes and *EE* might be influenced and it is often not possible to remove the non-entrapped fluorescence dye completely [11]. Thus, methods have been described to determine *EE* without removing the non-entrapped cargo. Oku et al. described a method for the determination of the trapped volume of liposomes using cobalt (Ⅱ) ions, which quench the fluorescence of accessible calcein by chelation [12]. Zhang et al. developed a non-fluorescent method for a similar purpose by using homocarnosine as a marker. It shows a pH-sensitive ^1^H chemical shift in proton NMR in the presence of a pH gradient across the vesicle membrane or after addition of the chemical shift reagent TmDOTO^5−^ [13]. However, both methods are very time consuming and subject to errors that render them unfavorable for a screening method where hundreds of samples have to be tested. Moreover, the samples might be affected by the addition of reagents or the formation of a pH gradient.

Time-resolved fluorescence is used for measuring fluorescence intensity decays. The sample is exposed to a very short pulse of laser light that is shorter than the decay time of the sample. The intensity decay is recorded on a nanosecond timescale using a high-speed photon detection system. In contrast, the commonly used steady-state fluorescence measurements are performed with constant illumination and detection and represent an average over the intensity decays of the time-resolved method. Time-resolved fluorescence measurements can provide additional information about the chemical surroundings or the concentration of the fluorescence dye [14]. Collisional self-quenching fluorescence dyes like calcein show concentration-dependent lifetimes [15] because the likelihood of quenching an excited dye by a collision increases with the time after excitation and with concentration.

Hence, self-quenching reduces the fluorescence lifetime, τ, of calcein from 4 ns in a dilute solution to, e.g., 0.4 ns at 70 mM calcein [15]. This effect can be used to differentiate between highly concentrated calcein trapped in liposomes and non-entrapped calcein which becomes diluted as VPGs are diluted to obtain a liposomal dispersion.

Patel et al. developed a leakage assay for calcein-loaded vesicles which allowed the parallel quantification of free and entrapped calcein using a biexponential fit of calcein fluorescence decays and thus the correlation of efflux with local dye concentration [15]. They prepared the liposomes by extrusion [16]. Since extrusion typically resulted in rather low liposome concentrations, the concentration gradient necessary for efflux studies cannot be established by a simple dilution of the liposome dispersion. Instead, the non-entrapped calcein had to be removed by using a desalting column [15]. Based on the higher liposome concentration within VPGs obtained by DC homogenization, it appeared possible to determine the *EE* after dilution of VPGs to liposomes without removing the non-entrapped calcein. This was achieved by using the idea of Patel et al. to use a parallel quantification of free and entrapped calcein [15] to directly determine the *EE* of a calcein containing liposome dispersion obtained by dilution of DC-prepared VPGs.

Considering that DC allows very intense and thorough homogenization of a mixed sample, we investigated whether it could eliminate the need for prior lipid film preparation. Virtually all other techniques to produce cholesterol-containing liposomes require a prior co-dissolution of all ingredients in an organic solvent. For extrusion or sonication, the solvent is removed to establish a molecularly mixed lipid film on the wall of the container prior to adding buffer and downstream processing. Reverse phase evaporation is based on injecting the organic solution into buffer and removing the solvent afterwards [17]. Another recent strategy to produce drug loaded liposomes at reduced utilization of organic solvents is the supercritical fluid technology (SCF) with supercritical carbon dioxide (scCO_2_) [18]. Frederiksen et al. described the preparation of liposomes loaded with water-soluble compounds using the SCF technology with scCO_2_ and could reduce the amount of ethanol needed as a co-solvent [19]. However, phospholipids are known to have a rather poor solubility in scCO_2_ so that the addition of some organic solvents may still be required [20,21]. For cholesterol-free liposomes of L-α-dioleoyl phosphatidylcholine (DOPC), Otake et al. achieved an encapsulation efficiency of ~36% in a co-solvent free SCF preparation utilizing an improved supercritical reverse phase evaporation [22]. 

The hypothesis that DC is capable of generating molecularly homogeneous mixed liposomes without the prior dissolution in any organic solvents is tested using differential scanning calorimetry (DSC). This exploits the fact that lipids with different phase transitions that mix in one phase share a common characteristic melting peak at an intermediate temperature. When these lipids coexist in a dispersion without equilibrating at a molecular level, the result is a superposition of peaks representing different, individual local compositions. 

To summarize, in this study, we developed a method for *EE* determination using time-resolved fluorescence measurements of calcein-loaded liposomes without removing the non-entrapped calcein. A very rapid screening method to find optimal lipid concentrations for DC homogenization of various lipid compositions was developed which allows the preparation of VPGs and liposomes without the need to first prepare a molecularly dispersed lipid-mixture. Furthermore, economic aspects of liposome preparation were considered by introducing the new parameter encapsulation capacity (*EC*).

## 2. Materials and Methods

### 2.1. Materials

Hydrogenated egg phosphatidylcholine (EPC3; ≥98%), egg phosphatidylcholine (EPC; ≥96%), 1,2-dipalmitoyl-sn-glycero-3-phosphocholine (DPPC; ≥99%), 1,2-distearoyl-sn-glycero-3-phosphocholine (DSPC; ≥99%), and N-(Carbonyl-methoxypolyethylene glycol-2000)-1,2-distearoyl-sn-glycero-3-phosphoethanolamine, sodium salt (DSPE-PEG_2000_; ≥98%) were kindly provided by Lipoid GmbH (Ludwigshafen, Germany).

Calcein (product number: C0875; batch number: SHBM5832) and cholesterol (≥99%) were obtained from Sigma-Aldrich (St. Louis, MO, USA). Tris(hydroxymethyl)aminomethane (Tris; ≥99.9%) and ethylenediaminetetraacetic acid (EDTA; ≥99%) were obtained from Carl Roth GmbH (Karlsruhe, Germany). All other chemicals were obtained from Carl Roth GmbH (Karlsruhe, Germany) and were of analytical grade.

### 2.2. Methods

#### 2.2.1. In-Vial Lipid Film Preparation

Lipid films were prepared using a modified thin-film hydration method for each lipid mixture. The dry lipids were dissolved in a chloroform/methanol (2:1, *v*/*v*) mixture to obtain a stock solution of 200 mM lipid concentration. Aliquots from this stock solution were pipetted directly into 2 mL conical screw cap vials (Sarstedt AG & Co. KG, Nümbrecht, Germany, Type 72.693.005). The organic solvents were evaporated at 38–41 °C in a vacuum centrifuge (RVC 2–18, Martin Christ Gefriertrockungsanlagen GmbH, Osterode, Germany) with an attached vacuum pump (MZ 2C, Vacuubrand GmbH + Co KG, Wertheim, Germany) for 4–8 h until a dry lipid film was formed. To ensure complete evaporation of the organic solvent, the lipid films were dried in a vacuum desiccator with a high-vacuum pump (RD 4, Vacuubrand GmbH + Co KG, Wertheim, Germany) for at least 2 h.

#### 2.2.2. Preparation of Liposomes by Dual Centrifugation

Samples were prepared by the dual centrifuge (DC) ZentriMix 380 R (Andreas Hettich GmbH & Co. KG, Tuttlingen, Germany). All samples for the liposome screenings were prepared in 2 mL conical screw cap vials, each with 600 mg zirconium oxide beads with a diameter of 1.4–1.6 mm (SiLibeads^®^ Type ZY-S, Sigmund Lindner GmbH, Germany), the lipid film, and calcein buffer (60 mM, 10 mM tris, 0.5 mM EDTA, pH 8.5) as the aqueous phase. Liposome preparation by DC was also conducted with lipid mixtures without lipid film preparation. Samples for liposome preparation by DC without lipid film preparation were prepared by weighting the dry lipid components, the zirconium oxide beads, and the aqueous phase directly into the vial. The settings for the preparation by DC without lipid film preparation were the same as described for the preparation of liposomes with lipid film preparation by DC. The standard settings for liposome preparation in DC were 2350 rpm, 30 min, and 20 °C. The resulting VPG was diluted to a liposomal suspension with tris buffer (10 mM tris buffer containing 0.5 mM EDTA, pH 8.5) in the ratio 2:1 (*v*/*v*) by DC (1500 rpm, 2 min, 20 °C). For all lipid mixtures, a batch size of 100 mg vesicular phospholipid gel (VPG) was prepared to standardize the influence of the beads and wetting of the vial surface. According to the lipid concentration used (2.5–85%), 2.5–85 mg lipid mixture and corresponding to 97.5–15 µL of aqueous buffer were used for the preparation of each VPG. The ratio of lipid to aqueous buffer in the VPG is given as lipid concentration (*m*/*v*). Due to a density close to 1 g/mL for both phospholipids and buffer, we approximate for convenience that 10 mg lipid per mL dispersion represents 1% in mass percent.

#### 2.2.3. Time-Resolved Fluorescence Measurements

The liposome suspension after preparation and redispersion in DC was diluted 1:5000 with tris buffer (4 µL liposome suspension + 1996 µL tris buffer; thereof 200 µL + 1800 µL tris buffer). Time-resolved fluorescence measurements were conducted at the fluorescence lifetime spectrometer FluoTime 100 (PicoQuant, Berlin, Germany). The excitation source was a modular laser diode (LDH-P-C-470) with a wavelength of 470 nm (±10 nm), pulsed at a repetition rate of 20 MHz. The laser was operated with the laser driver PDL 800-D (PicoQuant, Berlin, Germany). Decay curves were recorded at 515 nm wavelength by using time-correlated single photon counting (TCSPC). For each sample, the measurement conditions were set to a peak count of at least 10,000 photons by varying the transmission (0.1–1%) and the measuring period (20–50 s). Fluorescence decay curves were fitted with FluoFit software (PicoQuant, Berlin, Germany) by a biexponential function.

#### 2.2.4. Liposome Size and Size Distribution

The intensity-weighted hydrodynamic diameter (Z-average) and polydispersity index (PDI) of prepared liposomes were determined by DLS (Zetasizer Nano-ZS Malvern Instruments, Worcestershire, UK). Liposomal suspensions were diluted in tris buffer (viscosity: 0.8749 mPa·s; refractive index: 1.330) and the attenuator was set automatically to achieve a count rate of 150–250 kcps. Three measurements with an automatically selected number of scans (10–17 scans, 10 s/scan) were taken at 25 °C with a backscattering measurement at an angle of 173°.

#### 2.2.5. Differential Scanning Calorimetry

DSC experiments were performed with a VP-DSC (Malvern Instruments, Inc., Northampton, NC, USA). Liposomes for DSC experiments were loaded with tris buffer instead of calcein buffer. The sample cell was loaded with 1 mM liposomes diluted in 10 mM tris buffer. For the reference cell, plain tris buffer was used. Scans were run from 25–75 °C with a scanning rate of 60 K/h.

### 2.3. Statistical Analysis

The data are presented as mean ± standard deviation (SD) of three independent experiments.

DSC experiments are illustrated as a combined thermogram of single scans for each sample.

## 3. Results

### 3.1. Determination of the EE Using Time-Resolved Fluorescence

To produce a liposome dispersion from a VPG made in calcein buffer, the latter has to be diluted by, for example, a factor of 5000, which results in a dispersion containing two populations of calcein. Entrapped calcein remains at 60 mM, resulting in a fluorescence lifetime of *τ_E_* ≈ 0.4 ns. Non-entrapped (i.e., free) calcein is diluted strongly enough to eliminate self-quenching so that its lifetime, *τ_F_* = 4 ns. Hence, the overall fluorescence decay, *F(t)* of the dispersion, shows, to a good approximation, a biexponential behavior where the amplitudes *B_E_* and *B_F_* quantify the amounts of entrapped and free calcein per total volume:

(1) $$F(t)=B_{E}^{}\exp\left\{-\frac{t}{\tau_{E}}\right\}+B_{F}\exp\left\{-\frac{t}{\tau_{F}}\right\}$$

**Figure 1:**
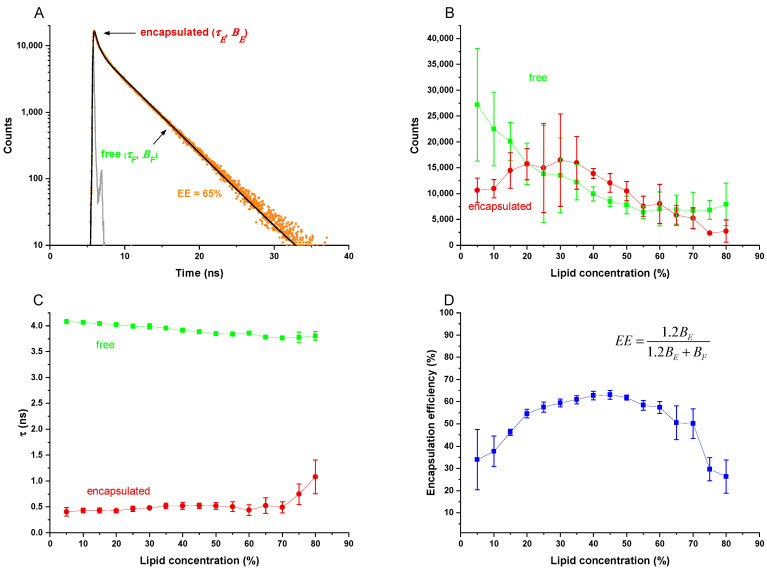
Time-resolved fluorescence decay for direct determination of the *EE* using the example of EPC/Chol 55/45 (mol/mol) liposomes. (**A**): Time-resolved fluorescence decay curve for the instrument response function (dark gray) and calcein-loaded EPC/Chol 55/45 (mol/mol) liposomes with 45% lipid concentration in the vesicular phospholipid gel during DC. The biexponential fit is shown as a black line. The *EE* is calculated from the corresponding pre-exponential factors (according to formula shown in (**C**)) and is indicated in the plot. (**B**): Pre-exponential factors of encapsulated and free calcein (proportional to concentrations of free and encapsulated calcein [15]) depending on the lipid concentration in the VPG during preparation by DC. (**C**): Fluorescence lifetime data of encapsulated and free calcein (simultaneous determination) depending on the lipid concentration in the VPG during preparation by DC. (**D**): Calculated *EE* using Formula (2) plotted against the lipid concentration in the VPG during preparation by DC. Data presented as mean ± SD, *n* = 3.

Figure 1A shows a typical fluorescence decay curve, *F(t)*, as obtained for calcein-loaded EPC/Chol 55/45 (mol/mol) liposomes prepared from a VPG of 45% lipid, along with the biexponential fit curve and the instrument response function. Figure 1B,C compile the fit parameters for liposomes of this composition as a function of the lipid content during DC. As expected, there is no exchange between entrapped and free dye after lipid formation and dilution. Thus, the two fluorescence lifetimes remain essentially constant, independently of the lipid concentration upon DC (Figure 1C). The preexponential factor of the free dye decreases gradually as more lipid is available to entrap dye molecules (Figure 1B).

Analogous to the standard definition of dye leakage [15], we obtain for the encapsulation efficiency, *EE*:

(2) $$EE\equiv \frac{c_{E}}{c_{E}+c_{F}}=\frac{1.2B_{E}}{1.2B_{E}+B_{F}}$$

The empiric factor of 1.2 corrects for very weak static quenching, which renders a small fraction of the entrapped dye undetected in terms of *B_E_* [15]. It expresses the finding that for any sample with constant total dye concentration but progressing leakage, i.e., *B_F_* increasing at the expense of *B_E_*, the sum *B_F_* + 1.2 *B_E_* remains constant.

Figure 1D shows the resulting *EE* as a function of the lipid concentration indicating a broad plateau of about 50% of all dye being entrapped upon centrifugation at about 30–70% of lipid.

### 3.2. DC without Prior Lipid Film Preparation from Organic Solvent

The data above were obtained by running the DC homogenization with films of lipids pre-mixed in organic solvent. Here, we tested whether DC homogenization eliminates the need for this time-consuming lipid film preparation and thus also eliminates the need for organic solvents.

**Figure 2:**
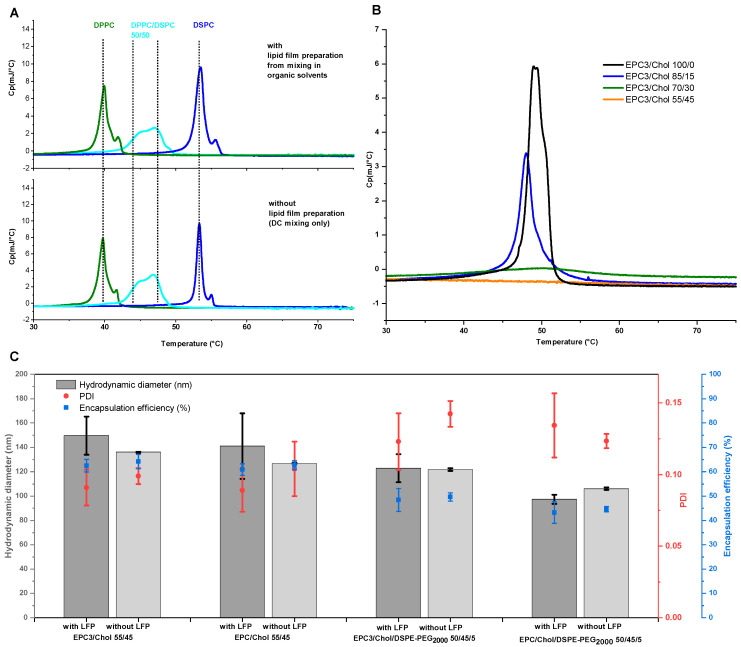
(**A**): Differential scanning calorimetry results for the investigation of the influence of lipid film preparation for the preparation of DPPC (green), 50/50 (mol/mol) DPPC/DSPC (cyan), and DSPC (blue) liposomes. Samples were prepared by DC (2350 rpm, 30 min, 20 °C) with 100 mg VPG batch sizes and lipid concentration in VPG of 61.5%. Following 2:1 (*v*/*v*) redispersion at 1500 rpm, 2 min, 20 °C with tris buffer. The concentration of each sample (DPPC, DPPC + DSPC for 50/50 (mol/mol) and DSPC) was 1 mM. The scanning range was from 25–75 °C at a heating rate of 60 K/h. (**B**): Differential scanning calorimetry results of EPC3 liposomes containing different amounts of Chol (with decreasing peak: 0%, 15%, 30%, and 45% Chol). Liposomes prepared without lipid film preparation to show a molecular dispersed mixture of EPC3 with cholesterol after preparation by DC (2350 rpm, 30 min, 20 °C) at 40% lipid concentration with 100 mg VPG batch sizes and 2:1 (*v*/*v*) redispersion at 1500 rpm, 2 min, 20 °C with Tris. The concentration of each sample was 1 mM (EPC3 + Chol). Black line: EPC3/Chol 100:0 (mol/mol). Blue line: EPC3/Chol 85:15 (mol/mol). Green line: EPC3/Chol 70:30 (mol/mol). Orange line: EPC3/Chol 55:45 (mol/mol). The scanning range was from 25–75 °C at a heating rate of 60 K/h. (**C**): Comparison of liposomes prepared with and without lipid film preparation. Lipid film preparation was performed to ensure a molecularly dispersed mixture of the lipid components. For EPC3/Chol 55/45, a molecularly dispersed lipid blend was used instead of lipid film preparation. Samples without lipid film preparation were prepared by weighing the lipid components directly into the vial. DC settings: 2350 rpm, 30 min, 20 °C, and 40% lipid concentration, 2:1 (*v*/*v*) redispersion of the VPG with Tris buffer by DC at 1500 rpm, 2 min, 20 °C. Following lipid mixtures were compared, each with and without lipid film preparation (LFP): EPC3/Chol 55:45 (mol/mol), EPC/Chol 55:45 (mol/mol), EPC3/Chol/DSPE-PEG2000 50:45:5 (mol/mol), EPC/Chol/DSPE-PEG2000 50:45:5 (mol/mol). Data presented as mean ± SD, *n* = 3.

DSC is a method that distinguishes molecularly dispersed mixtures of lipids from partially de-mixed, heterogeneous samples. While DSPC shows a sharp phase transition at 55.3 °C [23] and DPPC at 41.4 °C [23], mixtures of these lipids exhibit an isomorphous phase behavior with broader transitions with peaks at intermediate temperatures [24]. Figure 2A shows overlays of DSC diagrams for liposomes made from DPPC, DSPC, and the mixture (50/50 (mol/mol)) of both by DC with and without prior lipid film formation. Samples prepared by DC after a lipid film preparation were compared with samples where appropriate amounts of dry powders of DPPC and DSPC were added to buffer and ceramic beads in a DC vial, followed by the dual centrifugation procedure. Any incomplete mixing on the molecular level would cause a broadening of the transition due to molecular interactions in molecular environments with lower DSPC content (lower transition temperature range) and higher DSPC content (higher Tm-range). Such a broadening was not observed experimentally, which also indicates that dual centrifugation without prior mixing of the lipid components in organic solvent results in a homogeneous and molecularly dispersed lipid mixture.

The fact that the peaks are not fully monomodal can be explained by the fact that DC homogenization usually results in a mixture of multilamellar and unilamellar liposomes [2,7] and that multilamellar phospholipid structures show a highly cooperative behavior during the phase transition, which results in a narrower T_m_ peak [25]. Furthermore, smaller (unilamellar) vesicles show a phase transition at lower temperatures due to the increased curvature [25,26].

To investigate if lipid film formation can also be omitted if the desired liposomes contain cholesterol, mixtures of hydrogenated egg PC and increasing ratios of cholesterol without the formation of a lipid film were used in the DC homogenization process. DSC of the resulting liposomes showed that the pure hydrogenated egg PC has a clear and pronounced phase transition slightly below 50 °C since hydrogenated egg PC consists mainly of PC species with C16 and C18-fatty acid chains [27].

When increasing the cholesterol content, the peak becomes much smaller, while the phase transition temperature does not substantially change. A proportion of 30% cholesterol or more caused a complete disappearance of the peak, showing that the lipid bilayers of the liposomes obtained by DC without lipid film hydration are mixed in a molecularly dispersed manner. It is well known that phospholipid containing vesicles show a cholesterol-induced broadening and reduction of cooperativity during the phase transition [28]. The enthalpy decreases with increasing cholesterol concentrations and disappears at about 35–50 mol% [29,30].

Finally, DC homogenization of lipid blends with or without prior preparation of a molecularly dispersed lipid mixture in organic solvent (lipid film) also resulted in liposomes with virtually equal size, size distribution, and entrapment efficiencies (Figure 2C). Lipid film preparation only allowed for slightly higher homogeneity as indicated by lower standard deviations of these values. All tested lipid mixtures contained cholesterol as well as hydrogenated or non-hydrogenated PC, DSPE-PEG-2000, or no-PEG-component.

Taken together, the shear forces provided by the DC process are obviously high enough to mix the phospholipids and cholesterol in a molecularly dispersed manner.

### 3.3. Liposome Screening Profiles

**Figure 3:**
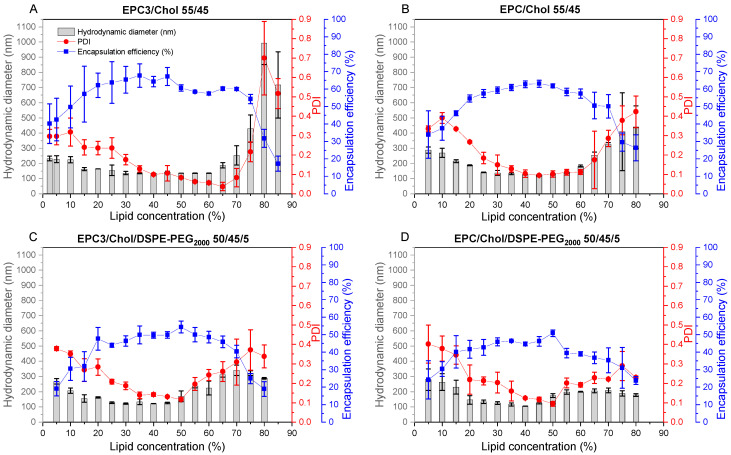
Liposome screening graphs show the hydrodynamic diameter, PDI, and *EE* as a function of the lipid concentration in the VPG during preparation by DC. DC settings: 2350 rpm, 30 min, 20 °C, and 2:1 (*v*/*v*) redispersion of the VPG with Tris buffer by DC at 1500 rpm, 2 min, 20 °C. (**A**): EPC3/Chol 55:45 (mol/mol), (**B**): EPC/Chol 55:45 (mol/mol), (**C**): EPC3/Chol/DSPE-PEG2000 50:45:5 (mol/mol), (**D**): EPC/Chol/DSPE-PEG2000 50:45:5 (mol/mol). Data presented as mean ± SD, *n* = 3.

To identify optimal conditions for the preparation of VPGs and subsequently liposomes, increasing concentrations of different lipid blends in 60 mM calcein solution were homogenized by DC. Since the dual centrifuge used in this study is able to process 40 samples at the same time, up to 13 different concentrations (in triplicates) can be processed in one 30 min run. The resulting VPGs were diluted in the ratio 2:1 with buffer and subjected to another short, 2 min treatment in the dual centrifuge at reduced speed for complete redispersion. The resulting liposomes were further diluted within a cuvette and *EE*, size, and PDI values were measured as described above (Figure 3). The results were presented in a “liposome screening graph” (Figure 3). The procedure described here establishes such a liposome screening diagram within one day.

To test the applicability of this approach, liposome screening diagrams of four different lipid mixtures were generated. All lipid blends contained 45% cholesterol, hydrogenated or non-hydrogenated egg phosphatidylcholine, and DSPE-PEG_2000_ or no PEG component.

All liposome screening graphs were found to show similar courses for the curves. For liposomes obtained from VPGs produced at a lipid concentration increasing from close to 0 to about 25%, the hydrodynamic diameter and PDI of the liposomes decreased, while *EE* increased. A further increase of the lipid content upon DC from about 25 to 70% reveals a plateau of all three parameters. Finally, as the lipid concentration is increased from about 70% to 80%, hydrodynamic diameter and PDI increased and *EE* generally decreased again.

For pharmaceutical applications of certain lipid mixtures, the protocol yielding a minimal hydrodynamic diameter, a low PDI, and high *EE* is of particular interest. The data should be as reproducible as possible and therefore should have a small standard deviation. The results for the lipid concentration, where the most optimal results were achieved according to the criteria described, are presented in Table 1.

### 3.4. Investigation of the Influence of PEG on DC-Made Liposome Characteristics

**Figure 4:**
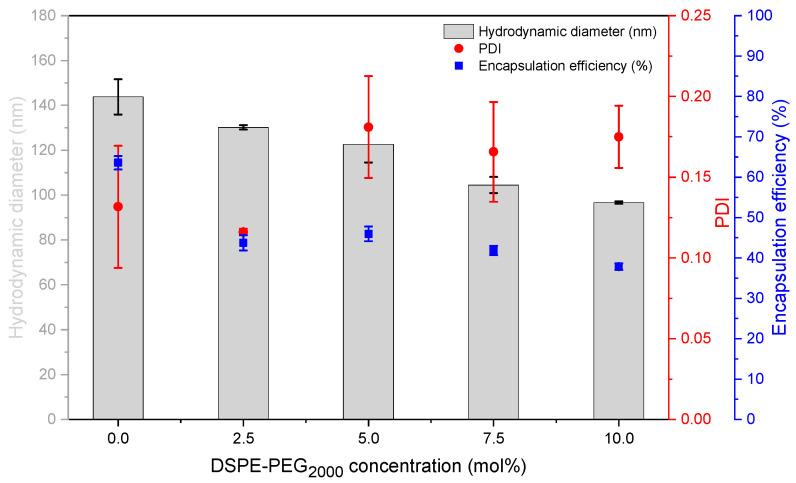
Influence of the DSPE-PEG_2000_ concentration on characteristics of DC-made EPC/Chol liposomes. With increasing DSPE-PEG_2000_ concentration, the EPC concentration was reduced accordingly, starting with EPC/Chol/ DSPE-PEG_2000_ 55/45/0 (mol/mol) up to EPC/Chol/DSPE-PEG_2000_ 45/45/10 at 10 mol% DSPE-PEG_2000_. DC settings: 2350 rpm, 30 min, 20 °C, and 40% lipid concentration in the VPG, 2:1 (*v*/*v*) redispersion of the VPG with Tris buffer by DC at 1500 rpm, 2 min, 20 °C. Data presented as mean ± SD, *n* = 3.

In a further step, the new liposome preparation and characterization techniques were used to investigate the influence of increasing PEG concentration on the characteristics of DC-made EPC/Chol liposomes (Figure 4) as an example for the screening of different lipid compositions. It was found that the size of the liposomes decreases with increasing PEG concentration. Inclusion of 2.5 mol% PEG-lipid into EPC/Chol 55/45 liposomes decreases *EE* by about 20%, but no significant trend of the *EE* was observed with increasing PEG content up to 10 mol%.

### 3.5. Uptake of Calcein after DC Homogenization of Empty VPGs

To address the issue of decreasing *EE* at very high lipid contents upon DC, we tested whether the very low water volume in these samples may be insufficient for the formation of closed liposomes in the first place. VPGs with increasing lipid concentrations were prepared by using only buffer with an addition of 150 mM NaCl but no calcein. Redispersion of the resulting VPGs with only very small amounts of calcein containing buffer resulted in vesicles in which significant amounts of calcein were entrapped when the lipid concentration upon DC was higher than about 70% (Table 2). This shows that closed liposomes allowing true entrapment of aqueous contents were not predominant in the highly concentrated VPGs. However, closed liposomes formed only upon dilution of the highly concentrated VPGs and entrapped the diluent.

## 4. Discussion

A first step in the design of a liposomal drug delivery system is the choice of its lipid constituents. The usage of saturated lipids and cholesterol renders liposomes particularly stable but may slow down drug release. PEG-grafted lipids may enhance circulation times by compromising the recognition of the liposomes by the immune system but may also hinder drug release by membrane fusion and cause adverse side effects. Consequently, there is no universal liposome formulation nor liposome composition and the production technology has to be adapted to a certain API, application route, and desired targeting and release characteristics.

### 4.1. DC Produces Liposomes from a Wide Variety of Lipid Compositions, Even without Lipid-Premixing in Organic Solvent

Formation of liposomes consisting of more than one lipid usually requires a molecularly dispersed lipid blend, especially when the poorly water-soluble cholesterol is part of the mixture. To get a so called “lipid film”, the lipids have to be dissolved in organic solvents (typically including the water-polluting chloroform) followed by a time consuming and careful removal of the solvents under vacuum, which involves the risk that lipid is lost by foaming over. Freeze drying from alcohol is also possible [1,6,31] but requires expensive equipment. SCF technologies reduce or eliminate the need for organic solvents for certain lipid formulations but require very expensive equipment [20], a complex optimization of the preparation conditions and are not suitable for bedside preparation. The efficient solubilization of the lipid mixture in scCO_2_ depends on various parameters, including pressure, temperature, time and the amount of a co-solvent [21].

Therefore, the finding that DC permits the formation of liposomes without the prior formation of a lipid film is a significant addition to the tool box of pharmaceutical liposome preparation. While the molecular mixing of different PC species could be expected during homogenization, the transfer of poorly water-soluble cholesterol from its crystalline form into the PC-bilayer was surprising. One explanation for that is the previous finding by Hagedorn et al. that DC is also a powerful tool for efficient nano-milling of water-insoluble compounds in an aqueous environment which contains detergents [32]. The formation of nano-sized cholesterol crystals greatly enhances their surface area, which results in a strong increase of the water-solubility and the fast integration of cholesterol molecules within the PC-bilayers. This process was likely supported by PC, which might interact in a detergent-like manner and help to release cholesterol molecules from the nanocrystals.

### 4.2. Novel Screening Procedure Allows Tailoring the DC Protocol to the Specific Needs of a Given Lipid Formulation

With the ability to process 40 samples at once, DC turned out to be a rapid screening tool for lipid compositions and homogenization conditions to finally identify a suitable liposomal drug delivery system for a specific application. Detailed knowledge about optimal lipid blends in combination with optimal preparation conditions can later be used to produce liposomes in bigger batches either by DC (e.g., for animal experiments or for bedside preparation) or by related means such as HPH. Furthermore, it was found that entrapping efficiency of the marker molecule calcein can be determined by time resolved fluorescence spectroscopy of the diluted liposomes without removing of the non-entrapped marker. This is very helpful in establishing a rapid screening procedure.

The methods typically used to determine *EE* require the time consuming and error prone separation of the non-trapped drug or marker molecules, e.g., by using desalting- or SEC-columns [1]. The method developed here is based on the fluorescence dye calcein, which is widely in use as a model for water soluble APIs. The new method is based on different fluorescence lifetimes of concentrated calcein inside the liposomes (0.4 ns) and of the non-entrapped calcein (4 ns) after dilution of the resulting VPGs (1:5000), which can individually be determined by time-resolved fluorescence spectroscopy. This technique is perfectly applicable here since liposomes made by DC or HPH typically have high *EE* values for water-soluble molecules. Thus, the ratio of trapped to free calcein is high enough to allow simultaneous counting of photons belonging to the fluorescence decay curves characterized by the different lifetimes (*τ_F_* and *τ_E_*) with high accuracy (compare Figure 1A,C).

Patel et al. showed that the longer fluorescence lifetimes of diluted calcein molecules of 4 ns can be measured up to 1 mM of calcein [15]. Since the calcein concentrations after dilution of the VPGs were much lower (below 10 µM), the new method is also expected to be applicable to liposomal formulations with about 100-times lower *EE*-values, which includes most liposomal formulations. In addition to this new method’s speed and capability of rapid screening, it is of particular advantage that the samples only have to be diluted prior to *EE* determination, which avoids the risk of liposome destruction during removing of non-entrapped molecules.

Another useful finding is that the same samples can also be used for DLS measurements (size and size distribution), using the same cuvette by dilution with additional buffer, which saves additional materials and time. Thus, the preparation as well as the determination of *EE* values, size, and size distribution of 40 liposomal formulations with different lipid compositions is now possible within one working day. The drawback of this method is that the equipment for time-resolved fluorescence spectroscopy is not available in many laboratories.

The results of screening a certain lipid composition were summarized in a liposome screening diagram, showing size, size distribution (PDI), and *EE* values for a wide range of lipid concentrations (2.5–85%) at a glance. Liposome screening diagrams are not restricted to a certain lipid composition. Instead of varying the concentration of a defined lipid composition, one can also vary the API content or the composition itself (as done for investigating of the influence of DSPE-PEG2000 (Figure 4)).

### 4.3. Lipid Concentration Determined Vesicle Size and Size Distribution

The liposome screening diagrams of the investigated phosphatidylcholine (PC)/Chol mixtures are similar and characterized by a decrease of size and narrower size distribution (lowered PDI) with increasing lipid concentration, followed by a concentration range in which the size and PDI are minimal. At higher lipid concentrations of about 65%, size and size distribution increase again.

The decrease of size and size distribution with increasing lipid concentrations can be explained by the increasing viscosity of the lipid mixtures, which allows the introduction of more energy during the DC process. The increase of size and PDI at very high lipid concentrations, for which a very high viscosity can also be assumed, can be explained by the presence of insufficient water to completely hydrate the membranes (discussed in detail below).

For each lipid mixture, a certain minimal liposome size seems to be a typical characteristic of a certain lipid composition which is reached between 25 and 60% lipid concentration. Even with longer homogenization times, this minimal size cannot further be reduced [1]. This “homogenization limit” is related to the “grinding limit”observed during nano-milling of poorly soluble drugs by DC [33]. The grinding limit represents an equilibrium between crystal downsizing and reformation of smaller crystals to larger ones. In analogy to that, the homogenization limit might be characterized by an equilibrium between the formation of small bilayer fragments from larger vesicles by disruption and correspondingly smaller vesicle formation as well as the reformation of larger vesicles from two or more of the smaller fragments.

The process of reformation to larger vesicles is likely supported by the close vicinity of the bilayer fragments within the highly concentrated VPGs, and by the higher energy demand which is necessary to form smaller vesicles with a narrower curvature [2]. This theory is supported by the finding that liposome size will not further decrease with increasing lipid concentrations, but the PDI will, showing that there is still an ongoing disruption of the small particles, although the average size remains the same. Previous studies have shown that liposome sizes decrease with increasing ratios of DSPE-PEG_2000_ [34,35,36] and that PEG-containing liposomes are more spherical and unilamellar [34,37]. It was discussed that the large hydrophilic head group of PEG lipids might cause a steric repulsion between the different lipid layers and thus results in liposomes with smaller size and reduced numbers of lamellae [34]. In line with that, we also showed a decrease in the size of DC-made EPC/Chol liposomes with increasing amounts of PEG lipids (Figure 4). Furthermore, the interval of minimal vesicle sizes in the liposome screening diagrams (Figure 3) shifted toward lower lipid concentrations when adding DSPE-PEG_2000_ to the lipid blends (compare Figure 3A–D), which can be explained by the higher demand of water for the hydration of the PEG headgroups.

### 4.4. Liposomal Packing Accounts for 3 Characteristic Regions of EE as a Function of Lipid Content upon DC

**Figure 5:**
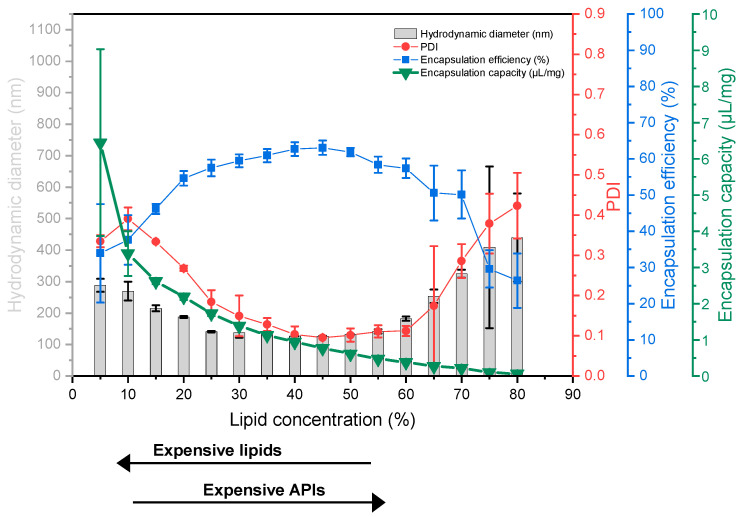
Selection of the optimal lipid concentration for liposome preparation by DC considering the economic factor encapsulation capacity (*EC*). The procedure is demonstrated by using the example of the liposome screening profile of EPC/Chol 55/45 (mol/mol), prepared by DC. *EC*, *EE*, size, and size distribution are plotted against the lipid concentration in the VPG during preparation by DC. *EC* is defined as the encapsulated aqueous phase relative to the mass of lipid contained in VPG during preparation by DC. Data presented as mean ± SD, *n* = 3.

The lipid concentration used for DC preparation of the VPGs and subsequently liposomes has great influence on the *EE* values (Figure 5). The EEs share a principal behavior including three characteristic regions: a quasi-linear increase up to ≈20% (Ⅰ), a plateau region up to roughly 70% (Ⅱ), and a region with decreasing *EE* above ≈70% (Ⅲ).

At low lipid concentrations, the volume fraction occupied by liposomes is low, so that liposome–liposome interactions are small. Each liposome entraps its core volume of drug solution and thus, the *EE* increases proportionally with the lipid concentration (region Ⅰ). Internal vesicles in oligovesicular or oligolamellar liposomes reduce the slope of this relationship, since they do not contribute to further entrapment, but rather fill space within the outermost liposome core with lipid.

It is important to recall that the mathematical limit for the densest packing of perfect, monodisperse spheres fills a volume fraction of 74%. Of course, the real system differs from this calculation in a number of ways. On the one hand, aqueous solutes are entrapped only in the aqueous core of the liposome, which is smaller than the outer volume by the volume filled by the lipids. This should limit *EE* of monodisperse, spherical liposomes to a value well below 74%. On the other hand, real VPGs comprise liposomes of different sizes and higher lamellarity, which increases the theoretical dense-packing limit. In line with these considerations, *EE*s of non-pegylated liposomes (cf. Figure 3A,B) approached maximal *EE* values of roughly 60% at about a lipid concentration of 20% (region Ⅰ), followed by a plateau with only minor changes of *EE* up to about 70% lipid (region Ⅱ). The larger volume required by PEG-lipids may explain the lower plateau values of *EE* in Figure 3C,D.

The decreasing *EE* in region Ⅲ, i.e., above ≈70% lipid, can be explained by an insufficient amount of water in the VPG to form stable, closed liposomes. With insufficient water, stacks of bilayers are likely to be formed that do not exhibit enclosed cores, and hence, cannot entrap a cargo. Only upon addition of additional buffer to the VPG, closed liposomal vesicles will be formed and thus enclose part of the aqueous medium added. It has been demonstrated that adding a small amount of calcein solution to a VPG of 80% lipid prepared without calcein leads to the partial entrapment of the “reconstitution medium” (*EE* > 50%). By contrast, VPGs produced at lipid contents below ≈70% (plateau region, Ⅱ) retain their original content and hardly take up any solution added upon reconstitution (Table 2). On the other hand, the liposome reconstitution process in region Ⅲ must reduce *EE* as reconstitution is performed with calcein-free buffer.

### 4.5. The Economic Aspect of DC-Homogenization—Introducing “Encapsulation Capacity”

At a first glance, the lipid concentration resulting in the lowest liposome sizes and narrow size distributions in combination with the highest *EE* values appears optimal and can typically be found around lipid concentrations of 40–50% used for DC homogenization. However, *EE* values take only the drug compounds into account, but not the amount of lipids which are part of the formulation. When it comes to liposomes made by DC or HPH, the lipid concentrations are rather high. Those high lipid concentrations automatically limit the amount of water (as well as the water-soluble drug compound) which can be entrapped even at high *EE* values. Thus, the “capacity” of DC-made liposomes to entrap a water-soluble drug is intrinsically low. Following that, we also considered the ratio of the water-soluble drug compound (here: calcein) to the amount of lipid used for entrapping the drug, defined as encapsulation capacity (*EC*), and indicated this with the unit (µL/mg). *EC* is calculated from the data obtained from the liposome screening experiments using Equation (3). *EC* is typically high at low lipid concentrations, but also depends on the *EE* values reached (Figure 5, green triangles).

Using *EC* allows the selection of the optimal lipid concentration from a more economic point of view, including the costs of the lipids and APIs. For example, if very expensive or rare APIs will be entrapped or only a very small number of liposomes is needed, one would select the lipid concentration resulting in the highest *EE* values in combination with the optimal particle sizes. Examples of these APIs are mRNA, siRNA, peptides, or rare natural products. If the lipid is the most expensive ingredient, or if higher amounts of liposomes are needed, one would select a lipid concentration which results in a higher *EC* value. Since the resulting liposomes tend to be rather large with a broader size distribution, those formulations can potentially be used for oral applications or as depot formulations (intraperitoneal injection, intramuscular injection, subcutaneous injection). A third scenario is an expensive lipid mixture, or the need for high amounts of liposomes, but with the necessity of small and uniform liposomes. Here, the optimal lipid-concentration for DC homogenization will be a compromise and will be found in between the previous examples, at the lowest possible lipid concentration which produces acceptable liposomes.

(3) $$EC=EE\times \frac{V\left(Calcein-buffer\;used\;as\;aqueous\;phase\right)\;}{m\left(Lipid-components\right)}$$

## 5. Conclusions

We present a fast, economic, and convenient screening procedure to optimize liposomes obtained from vesicular phospholipid gels (VPGs) with respect to encapsulation efficiency (*EE*), encapsulation capacity of the lipid (*EC*), and the size and size distribution (PDI) of the liposomes. Therefore, we developed a new method for the direct determination of the *EE* by using time-resolved fluorescence measurements, without the need to remove the non-entrapped calcein. Furthermore, we were able to show that lipid film preparation is not necessary for liposome preparation by DC, which additionally accelerates and simplifies the screening process.

Using this approach, favorable protocols were identified to produce VPGs of different lipid compositions via dual centrifugation (DC) and to optimize the preparation for the specific needs of a selected API (including si- or mRNA, hydrophilic and hydrophobic drugs), application route (intravenous, subcutaneous injection, oral, etc.), or release profile (including temperature-sensitive liposomes, etc.).

This renders DC a very promising technique to produce small batches of liposomal formulations as needed for personalized bedside production, small studies, or the development of VPGs that can later be scaled up by switching from DC to HPH technology.

## Figures and Tables

**Table 1:** Liposome characteristics for DC liposome screenings at selected optimal lipid concentrations. Data presented as mean ± SD, *n* = 3.

Lipid Mixture	Lipid Concentration (%)	Hydrodynamic Diameter (nm)	PDI	*EE* (%)
EPC3/Chol 55/45	60	136 ± 1	0.057 ± 0.008	57.4 ± 0.9
EPC/Chol 55/45	45	126 ± 2	0.095 ± 0.002	63.1 ± 2.0
EPC3/Chol/PEG 50/45/5	45	127 ± 5	0.131 ± 0.003	49.9 ± 1.9
EPC/Chol/PEG 50/45/5	40	106 ± 1	0.123 ± 0.005	44.6 ± 1.1

**Table 2:** The uptake of calcein of DC-made empty VPGs is demonstrated using liposomes made by DC with EPC3/Chol 55/45 (mol/mol). The VPG was prepared with 150 mM NaCl containing Tris buffer. Calcein was then added to the VPG and incubated for 5 min. The *EE* was measured by time-resolved fluorescence as described for the liposome screening procedure. Data presented as mean ± SD, *n* = 3.

Lipid Concentration during Preparation by DC	Lipid Concentration after Adding Calcein Buffer	Hydrodynamic Diameter (nm)	PDI	Encapsulation Efficiency (%)
80	60	1554 ± 374	0.50 ± 0.31	52.6 ± 2.9
80	40	1488 ± 329	0.69 ± 0.20	45.4 ± 1.9
60	40	155 ± 19	0.12 ± 0.10	8.1 ± 0.7
40	25	161 ± 15	0.22 ± 0.08	9.9 ± 2.2

## Data Availability

Not applicable.
